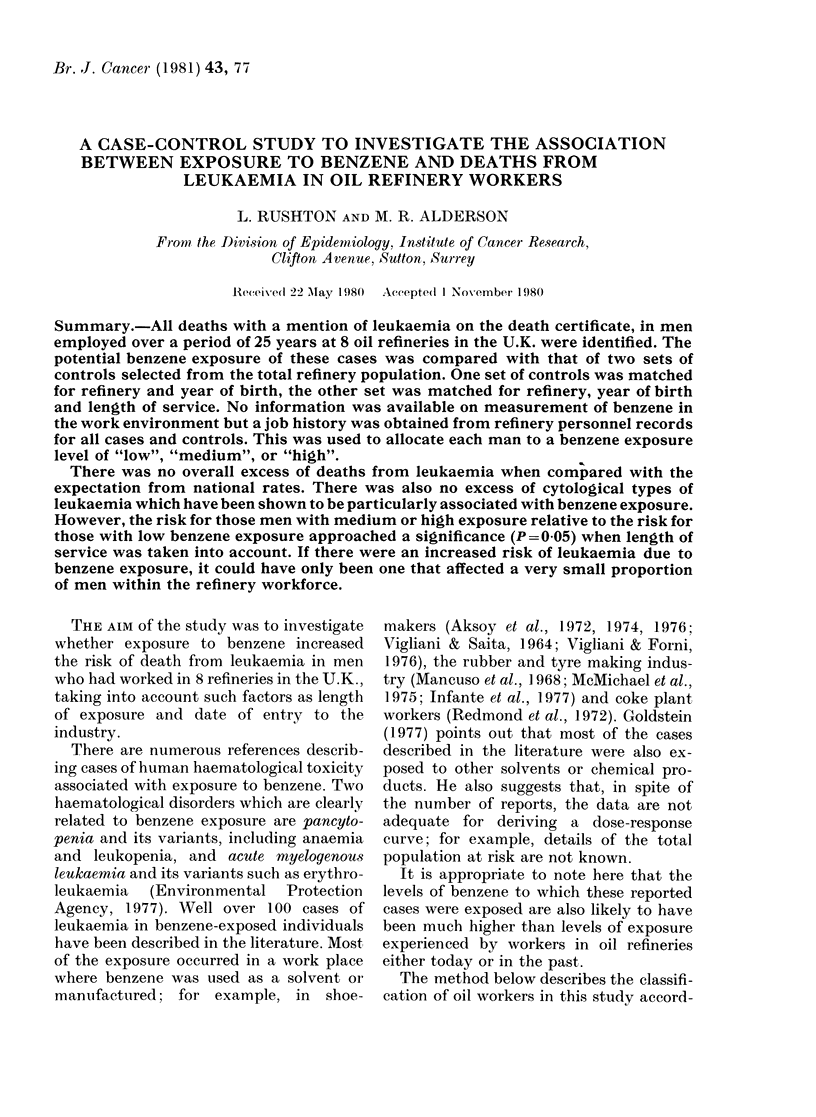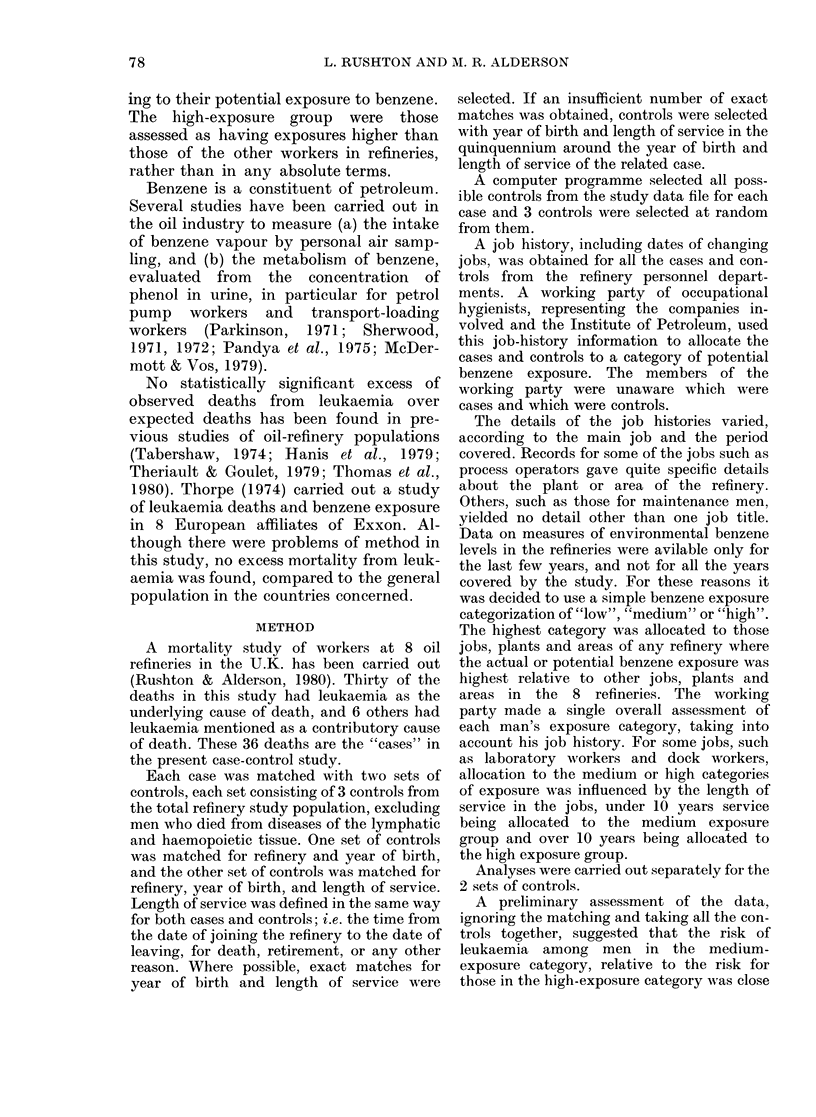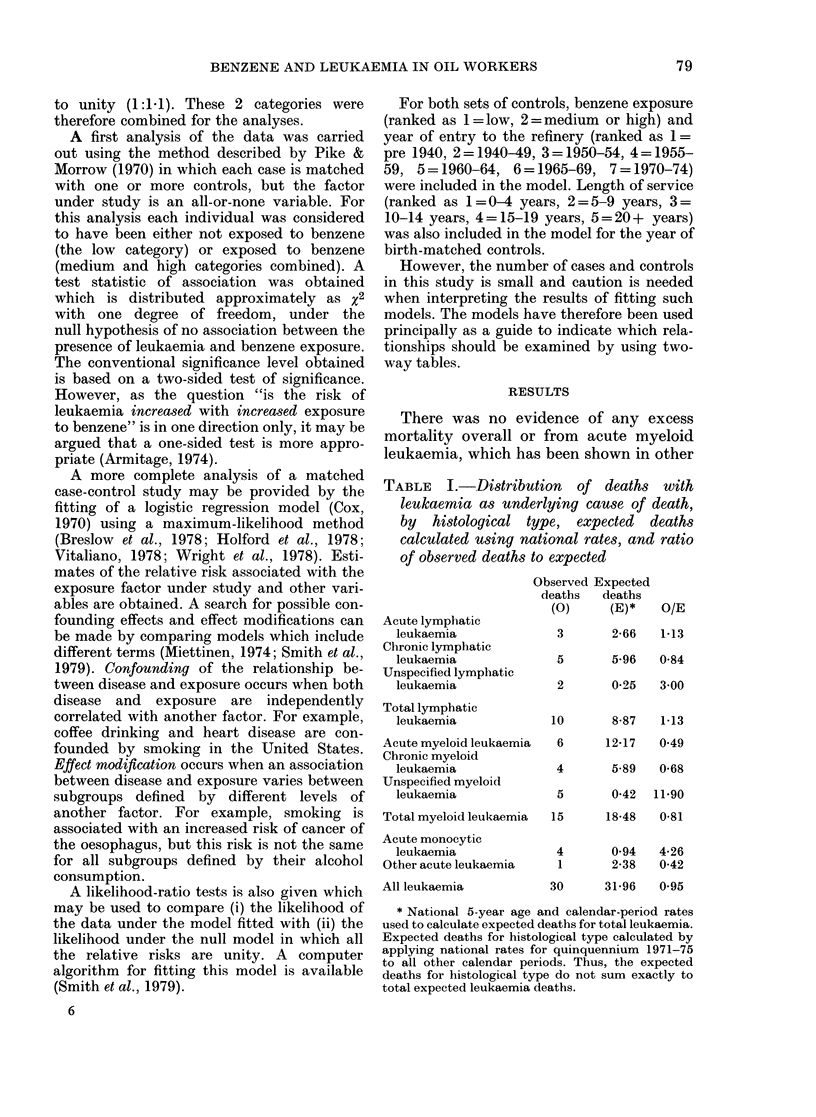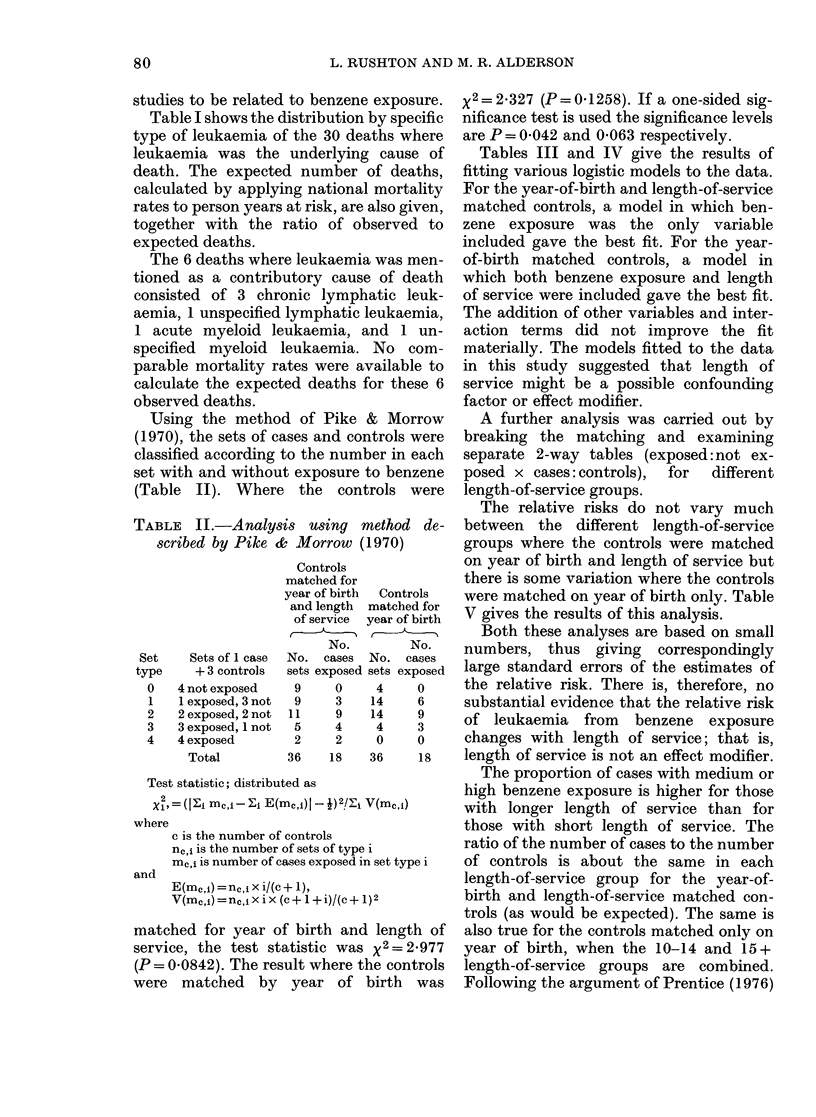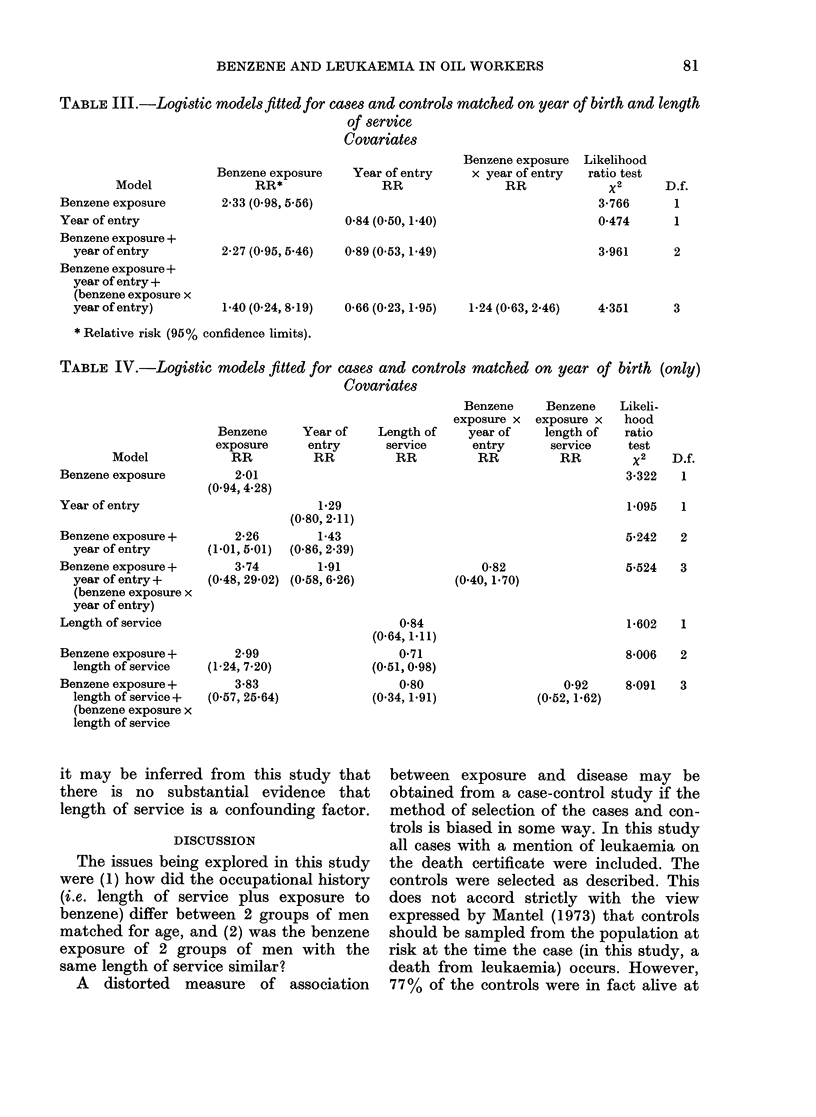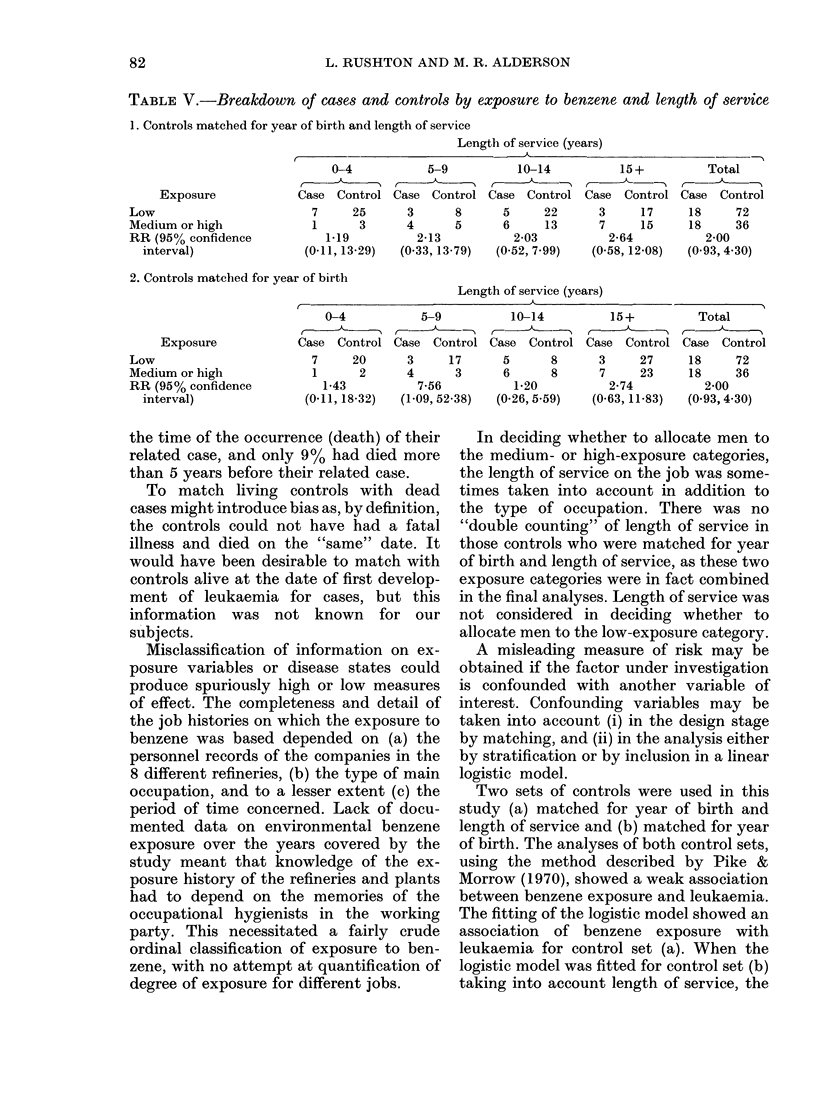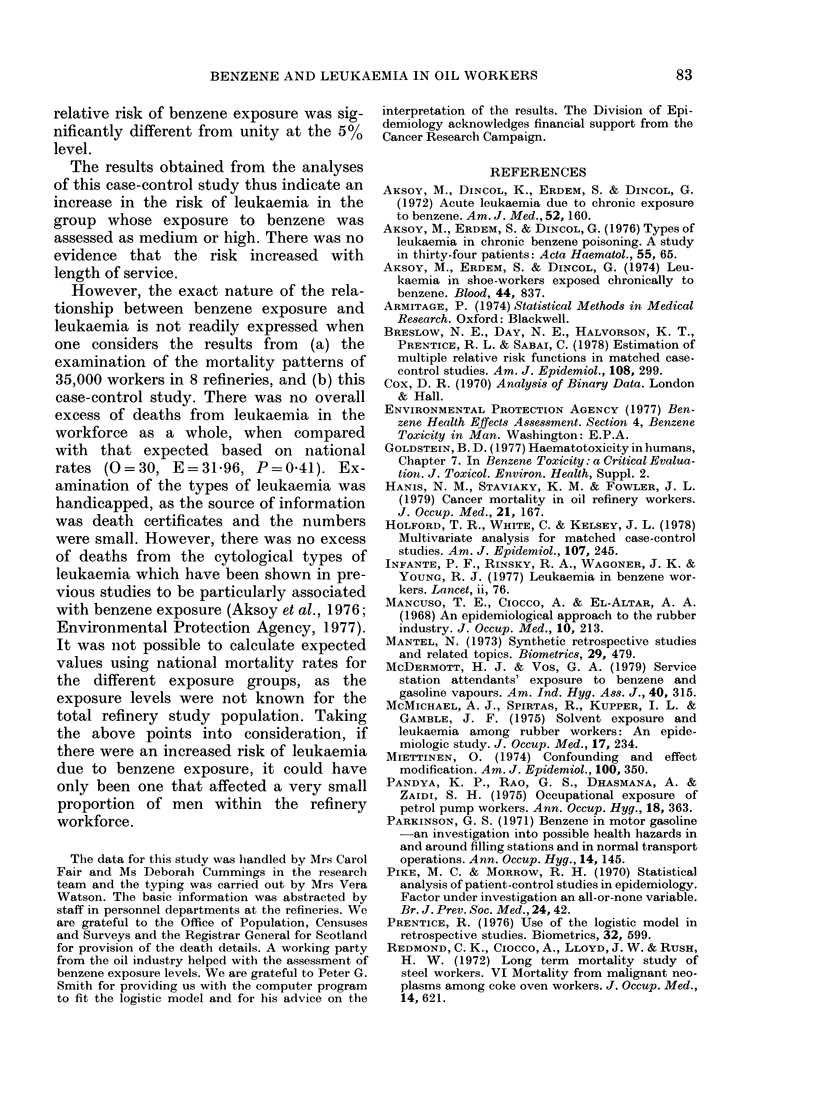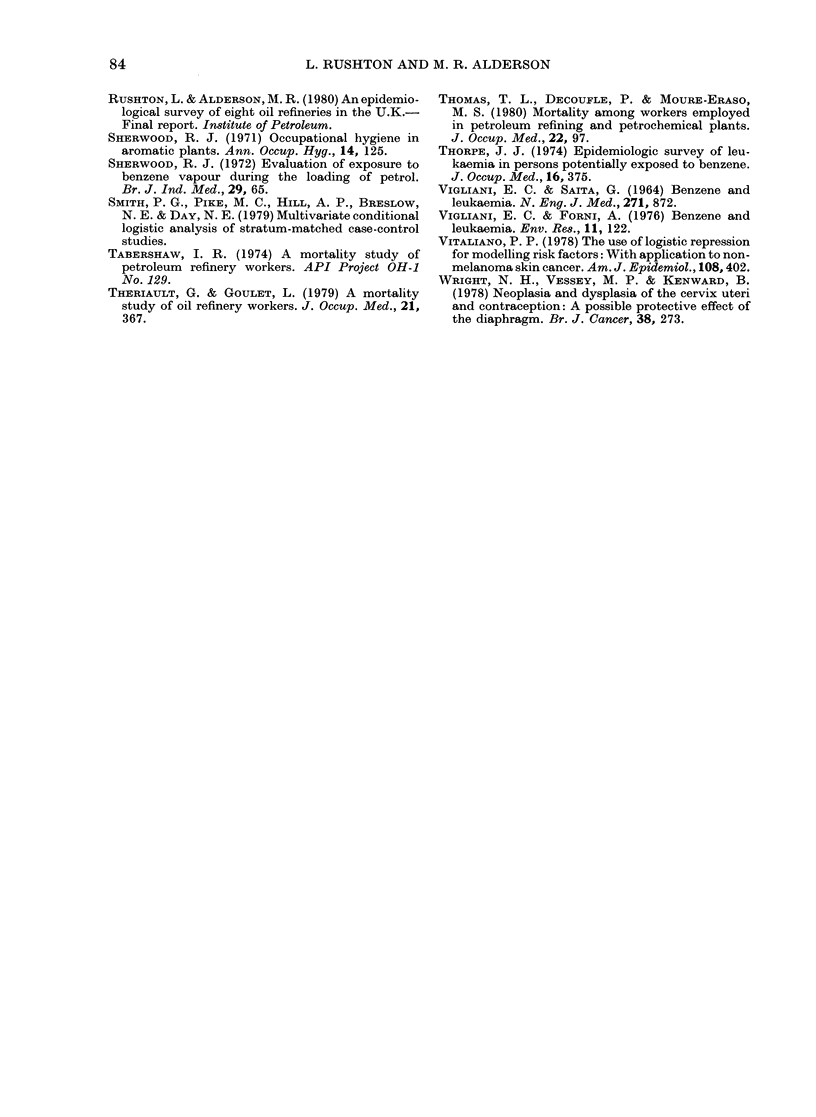# A case-control study to investigate the association between exposure to benzene and deaths from leukaemia in oil refinery workers.

**DOI:** 10.1038/bjc.1981.11

**Published:** 1981-01

**Authors:** L. Rushton, M. R. Alderson

## Abstract

All deaths with a mention of leukaemia on the death certificate, in men employed over a period of 25 years of 8 oil refineries in the U.K. were identified. The potential benzene exposure of these cases was compared with that of two sets of controls selected from the total refinery population. One set of controls was matched for refinery and year of birth, the other set was matched for refinery, year of birth nd length of service. No information was available on measurement of benzene in the work environment but a job history was obtained from refinery personnel records for all cases and controls. This was used to allocate each man to a benzene exposure level of "low", "medium", or "high". There was no overall excess of deaths from leukaemia when compared with the expectation from national rates. There was also no excess of cytological types of leukaemia which have been shown to be particularly associated with benzene exposure. However, the risk for those men with medium or high exposure relative to the risk for those with low benzene exposure approached a significance (P=0.05) when length of service was taken into account. If there were an increased risk of leukaemia due to benzene exposure, it could have only been one that affected a very small proportion of men within the refinery workforce.


*Br. J. Cancer* (1981) **43**, 77

### A CASE-CONTROL STUDY TO INVESTIGATE THE ASSOCIATION BETWEEN EXPOSURE TO BENZENE AND DEATHS FROM LEUKAEMIA IN OIL REFINERY WORKERS

L. RUSHTON AND M. R. ALDERSON

*From the Division of Epidemiology, Institute of Cancer Research, Clifton Avenue, Sutton, Surrey*

Received 22 May 1980 Accepted 1 November 1980

**Summary.—All deaths with a mention of leukaemia on the death certificate, in men employed over a period of 25 years at 8 oil refineries in the U.K. were identified. The potential benzene exposure of these cases was compared with that of two sets of controls selected from the total refinery population. One set of controls was matched for refinery and year of birth, the other set was matched for refinery, year of birth and length of service. No information was available on measurement of benzene in the work environment but a job history was obtained from refinery personnel records for all cases and controls. This was used to allocate each man to a benzene exposure level of "low", "medium", or "high".**

**There was no overall excess of deaths from leukaemia when compared with the expectation from national rates. There was also no excess of cytological types of leukaemia which have been shown to be particularly associated with benzene exposure. However, the risk for those men with medium or high exposure relative to the risk for those with low benzene exposure approached a significance ($P = 0{\cdot}05$) when length of service was taken into account. If there were an increased risk of leukaemia due to benzene exposure, it could have only been one that affected a very small proportion of men within the refinery workforce.**

THE AIM of the study was to investigate whether exposure to benzene increased the risk of death from leukaemia in men who had worked in 8 refineries in the U.K., taking into account such factors as length of exposure and date of entry to the industry.

There are numerous references describing cases of human haematological toxicity associated with exposure to benzene. Two haematological disorders which are clearly related to benzene exposure are *pancytopenia* and its variants, including anaemia and leukopenia, and *acute myelogenous leukaemia* and its variants such as erythroleukaemia (Environmental Protection Agency, 1977). Well over 100 cases of leukaemia in benzene-exposed individuals have been described in the literature. Most of the exposure occurred in a work place where benzene was used as a solvent or manufactured; for example, in shoemakers (Aksoy *et al.*, 1972, 1974, 1976; Vigliani & Saita, 1964; Vigliani & Forni, 1976), the rubber and tyre making industry (Mancuso *et al.*, 1968; McMichael *et al.*, 1975; Infante *et al.*, 1977) and coke plant workers (Redmond *et al.*, 1972). Goldstein (1977) points out that most of the cases described in the literature were also exposed to other solvents or chemical products. He also suggests that, in spite of the number of reports, the data are not adequate for deriving a dose-response curve; for example, details of the total population at risk are not known.

It is appropriate to note here that the levels of benzene to which these reported cases were exposed are also likely to have been much higher than levels of exposure experienced by workers in oil refineries either today or in the past.

The method below describes the classification of oil workers in this study according to their potential exposure to benzene. The high-exposure group were those assessed as having exposures higher than those of the other workers in refineries, rather than in any absolute terms.

Benzene is a constituent of petroleum. Several studies have been carried out in the oil industry to measure (a) the intake of benzene vapour by personal air sampling, and (b) the metabolism of benzene, evaluated from the concentration of phenol in urine, in particular for petrol pump workers and transport-loading workers (Parkinson, 1971; Sherwood, 1971, 1972; Pandya *et al.*, 1975; McDermott & Vos, 1979).

No statistically significant excess of observed deaths from leukaemia over expected deaths has been found in previous studies of oil-refinery populations (Tabershaw, 1974; Hanis *et al.*, 1979; Theriault & Goulet, 1979; Thomas *et al.*, 1980). Thorpe (1974) carried out a study of leukaemia deaths and benzene exposure in 8 European affiliates of Exxon. Although there were problems of method in this study, no excess mortality from leukaemia was found, compared to the general population in the countries concerned.

### METHOD

A mortality study of workers at 8 oil refineries in the U.K. has been carried out (Rushton & Alderson, 1980). Thirty of the deaths in this study had leukaemia as the underlying cause of death, and 6 others had leukaemia mentioned as a contributory cause of death. These 36 deaths are the "cases" in the present case-control study.

Each case was matched with two sets of controls, each set consisting of 3 controls from the total refinery study population, excluding men who died from diseases of the lymphatic and haemopoietic tissue. One set of controls was matched for refinery and year of birth, and the other set of controls was matched for refinery, year of birth, and length of service. Length of service was defined in the same way for both cases and controls; *i.e.* the time from the date of joining the refinery to the date of leaving, for death, retirement, or any other reason. Where possible, exact matches for year of birth and length of service were selected. If an insufficient number of exact matches was obtained, controls were selected with year of birth and length of service in the quinquennium around the year of birth and length of service of the related case.

A computer programme selected all possible controls from the study data file for each case and 3 controls were selected at random from them.

A job history, including dates of changing jobs, was obtained for all the cases and controls from the refinery personnel departments. A working party of occupational hygienists, representing the companies involved and the Institute of Petroleum, used this job-history information to allocate the cases and controls to a category of potential benzene exposure. The members of the working party were unaware which were cases and which were controls.

The details of the job histories varied, according to the main job and the period covered. Records for some of the jobs such as process operators gave quite specific details about the plant or area of the refinery. Others, such as those for maintenance men, yielded no detail other than one job title. Data on measures of environmental benzene levels in the refineries were avilable only for the last few years, and not for all the years covered by the study. For these reasons it was decided to use a simple benzene exposure categorization of "low", "medium" or "high". The highest category was allocated to those jobs, plants and areas of any refinery where the actual or potential benzene exposure was highest relative to other jobs, plants and areas in the 8 refineries. The working party made a single overall assessment of each man's exposure category, taking into account his job history. For some jobs, such as laboratory workers and dock workers, allocation to the medium or high categories of exposure was influenced by the length of service in the jobs, under 10 years service being allocated to the medium exposure group and over 10 years being allocated to the high exposure group.

Analyses were carried out separately for the 2 sets of controls.

A preliminary assessment of the data, ignoring the matching and taking all the controls together, suggested that the risk of leukaemia among men in the medium-exposure category, relative to the risk for those in the high-exposure category was close to unity (1:1·1). These 2 categories were therefore combined for the analyses.

A first analysis of the data was carried out using the method described by Pike & Morrow (1970) in which each case is matched with one or more controls, but the factor under study is an all-or-none variable. For this analysis each individual was considered to have been either not exposed to benzene (the low category) or exposed to benzene (medium and high categories combined). A test statistic of association was obtained which is distributed approximately as $\chi^2$ with one degree of freedom, under the null hypothesis of no association between the presence of leukaemia and benzene exposure. The conventional significance level obtained is based on a two-sided test of significance. However, as the question "is the risk of leukaemia *increased* with *increased* exposure to benzene" is in one direction only, it may be argued that a one-sided test is more appropriate (Armitage, 1974).

A more complete analysis of a matched case-control study may be provided by the fitting of a logistic regression model (Cox, 1970) using a maximum-likelihood method (Breslow *et al.*, 1978; Holford *et al.*, 1978; Vitaliano, 1978; Wright *et al.*, 1978). Estimates of the relative risk associated with the exposure factor under study and other variables are obtained. A search for possible confounding effects and effect modifications can be made by comparing models which include different terms (Miettinen, 1974; Smith *et al.*, 1979). *Confounding* of the relationship between disease and exposure occurs when both disease and exposure are independently correlated with another factor. For example, coffee drinking and heart disease are confounded by smoking in the United States. *Effect modification* occurs when an association between disease and exposure varies between subgroups defined by different levels of another factor. For example, smoking is associated with an increased risk of cancer of the oesophagus, but this risk is not the same for all subgroups defined by their alcohol consumption.

A likelihood-ratio tests is also given which may be used to compare (i) the likelihood of the data under the model fitted with (ii) the likelihood under the null model in which all the relative risks are unity. A computer algorithm for fitting this model is available (Smith *et al.*, 1979).

For both sets of controls, benzene exposure (ranked as 1 = low, 2 = medium or high) and year of entry to the refinery (ranked as 1 = pre 1940, 2 = 1940–49, 3 = 1950–54, 4 = 1955–59, 5 = 1960–64, 6 = 1965–69, 7 = 1970–74) were included in the model. Length of service (ranked as 1 = 0–4 years, 2 = 5–9 years, 3 = 10–14 years, 4 = 15–19 years, 5 = 20+ years) was also included in the model for the year of birth-matched controls.

However, the number of cases and controls in this study is small and caution is needed when interpreting the results of fitting such models. The models have therefore been used principally as a guide to indicate which relationships should be examined by using two-way tables.

### RESULTS

There was no evidence of any excess mortality overall or from acute myeloid leukaemia, which has been shown in other

TABLE I.—*Distribution of deaths with leukaemia as underlying cause of death, by histological type, expected deaths calculated using national rates, and ratio of observed deaths to expected*

| | Observed deaths (O) | Expected deaths (E)* | O/E |
|---|---|---|---|
| Acute lymphatic leukaemia | 3 | 2·66 | 1·13 |
| Chronic lymphatic leukaemia | 5 | 5·96 | 0·84 |
| Unspecified lymphatic leukaemia | 2 | 0·25 | 3·00 |
| Total lymphatic leukaemia | 10 | 8·87 | 1·13 |
| Acute myeloid leukaemia | 6 | 12·17 | 0·49 |
| Chronic myeloid leukaemia | 4 | 5·89 | 0·68 |
| Unspecified myeloid leukaemia | 5 | 0·42 | 11·90 |
| Total myeloid leukaemia | 15 | 18·48 | 0·81 |
| Acute monocytic leukaemia | 4 | 0·94 | 4·26 |
| Other acute leukaemia | 1 | 2·38 | 0·42 |
| All leukaemia | 30 | 31·96 | 0·95 |

* National 5-year age and calendar-period rates used to calculate expected deaths for total leukaemia. Expected deaths for histological type calculated by applying national rates for quinquennium 1971–75 to all other calendar periods. Thus, the expected deaths for histological type do not sum exactly to total expected leukaemia deaths.

studies to be related to benzene exposure.

Table I shows the distribution by specific type of leukaemia of the 30 deaths where leukaemia was the underlying cause of death. The expected number of deaths, calculated by applying national mortality rates to person years at risk, are also given, together with the ratio of observed to expected deaths.

The 6 deaths where leukaemia was mentioned as a contributory cause of death consisted of 3 chronic lymphatic leukaemia, 1 unspecified lymphatic leukaemia, 1 acute myeloid leukaemia, and 1 unspecified myeloid leukaemia. No comparable mortality rates were available to calculate the expected deaths for these 6 observed deaths.

Using the method of Pike & Morrow (1970), the sets of cases and controls were classified according to the number in each set with and without exposure to benzene (Table II). Where the controls were matched for year of birth and length of service, the test statistic was $\chi^2 = 2{\cdot}977$ ($P = 0{\cdot}0842$). The result where the controls were matched by year of birth was $\chi^2 = 2{\cdot}327$ ($P = 0{\cdot}1258$). If a one-sided significance test is used the significance levels are $P = 0{\cdot}042$ and $0{\cdot}063$ respectively.

TABLE II.—*Analysis using method described by Pike & Morrow* (1970)

| Set type | Sets of 1 case + 3 controls | Controls matched for year of birth and length of service: No. sets | Controls matched for year of birth and length of service: No. cases exposed | Controls matched for year of birth: No. sets | Controls matched for year of birth: No. cases exposed |
|---|---|---|---|---|---|
| 0 | 4 not exposed | 9 | 0 | 4 | 0 |
| 1 | 1 exposed, 3 not | 9 | 3 | 14 | 6 |
| 2 | 2 exposed, 2 not | 11 | 9 | 14 | 9 |
| 3 | 3 exposed, 1 not | 5 | 4 | 4 | 3 |
| 4 | 4 exposed | 2 | 2 | 0 | 0 |
| | Total | 36 | 18 | 36 | 18 |

Test statistic; distributed as

$$\chi^2_{1}, = (|\Sigma_i\, m_{c,i} - \Sigma_i\, E(m_{c,i})| - \tfrac{1}{2})^2 / \Sigma_i\, V(m_{c,i})$$

where c is the number of controls

$n_{c,i}$ is the number of sets of type i

$m_{c,i}$ is number of cases exposed in set type i and

$$E(m_{c,i}) = n_{c,i} \times i/(c+1),$$
$$V(m_{c,i}) = n_{c,i} \times i \times (c+1+i)/(c+1)^2$$

Tables III and IV give the results of fitting various logistic models to the data. For the year-of-birth and length-of-service matched controls, a model in which benzene exposure was the only variable included gave the best fit. For the year-of-birth matched controls, a model in which both benzene exposure and length of service were included gave the best fit. The addition of other variables and interaction terms did not improve the fit materially. The models fitted to the data in this study suggested that length of service might be a possible confounding factor or effect modifier.

A further analysis was carried out by breaking the matching and examining separate 2-way tables (exposed:not exposed × cases:controls), for different length-of-service groups.

The relative risks do not vary much between the different length-of-service groups where the controls were matched on year of birth and length of service but there is some variation where the controls were matched on year of birth only. Table V gives the results of this analysis.

Both these analyses are based on small numbers, thus giving correspondingly large standard errors of the estimates of the relative risk. There is, therefore, no substantial evidence that the relative risk of leukaemia from benzene exposure changes with length of service; that is, length of service is not an effect modifier.

The proportion of cases with medium or high benzene exposure is higher for those with longer length of service than for those with short length of service. The ratio of the number of cases to the number of controls is about the same in each length-of-service group for the year-of-birth and length-of-service matched controls (as would be expected). The same is also true for the controls matched only on year of birth, when the 10–14 and 15+ length-of-service groups are combined. Following the argument of Prentice (1976)

TABLE III.—*Logistic models fitted for cases and controls matched on year of birth and length of service*

| Model | Benzene exposure RR* | Year of entry RR | Benzene exposure × year of entry RR | Likelihood ratio test $\chi^2$ | D.f. |
|---|---|---|---|---|---|
| | *Covariates* | | | | |
| Benzene exposure | 2·33 (0·98, 5·56) | | | 3·766 | 1 |
| Year of entry | | 0·84 (0·50, 1·40) | | 0·474 | 1 |
| Benzene exposure + year of entry | 2·27 (0·95, 5·46) | 0·89 (0·53, 1·49) | | 3·961 | 2 |
| Benzene exposure + year of entry + (benzene exposure × year of entry) | 1·40 (0·24, 8·19) | 0·66 (0·23, 1·95) | 1·24 (0·63, 2·46) | 4·351 | 3 |

* Relative risk (95% confidence limits).

TABLE IV.—*Logistic models fitted for cases and controls matched on year of birth (only)*

| Model | Benzene exposure RR | Year of entry RR | Length of service RR | Benzene exposure × year of entry RR | Benzene exposure × length of service RR | Likelihood ratio test $\chi^2$ | D.f. |
|---|---|---|---|---|---|---|---|
| | *Covariates* | | | | | | |
| Benzene exposure | 2·01 (0·94, 4·28) | | | | | 3·322 | 1 |
| Year of entry | | 1·29 (0·80, 2·11) | | | | 1·095 | 1 |
| Benzene exposure + year of entry | 2·26 (1·01, 5·01) | 1·43 (0·86, 2·39) | | | | 5·242 | 2 |
| Benzene exposure + year of entry + (benzene exposure × year of entry) | 3·74 (0·48, 29·02) | 1·91 (0·58, 6·26) | | 0·82 (0·40, 1·70) | | 5·524 | 3 |
| Length of service | | | 0·84 (0·64, 1·11) | | | 1·602 | 1 |
| Benzene exposure + length of service | 2·99 (1·24, 7·20) | | 0·71 (0·51, 0·98) | | | 8·006 | 2 |
| Benzene exposure + length of service + (benzene exposure × length of service | 3·83 (0·57, 25·64) | | 0·80 (0·34, 1·91) | | 0·92 (0·52, 1·62) | 8·091 | 3 |

it may be inferred from this study that there is no substantial evidence that length of service is a confounding factor.

### DISCUSSION

The issues being explored in this study were (1) how did the occupational history (*i.e.* length of service plus exposure to benzene) differ between 2 groups of men matched for age, and (2) was the benzene exposure of 2 groups of men with the same length of service similar?

A distorted measure of association between exposure and disease may be obtained from a case-control study if the method of selection of the cases and controls is biased in some way. In this study all cases with a mention of leukaemia on the death certificate were included. The controls were selected as described. This does not accord strictly with the view expressed by Mantel (1973) that controls should be sampled from the population at risk at the time the case (in this study, a death from leukaemia) occurs. However, 77% of the controls were in fact alive at

TABLE V.—*Breakdown of cases and controls by exposure to benzene and length of service*

1. Controls matched for year of birth and length of service

| | Length of service (years) | | | | | | | | | |
|---|---|---|---|---|---|---|---|---|---|---|
| | 0–4 | | 5–9 | | 10–14 | | 15+ | | Total | |
| Exposure | Case | Control | Case | Control | Case | Control | Case | Control | Case | Control |
| Low | 7 | 25 | 3 | 8 | 5 | 22 | 3 | 17 | 18 | 72 |
| Medium or high | 1 | 3 | 4 | 5 | 6 | 13 | 7 | 15 | 18 | 36 |
| RR (95% confidence interval) | 1·19 (0·11, 13·29) | | 2·13 (0·33, 13·79) | | 2·03 (0·52, 7·99) | | 2·64 (0·58, 12·08) | | 2·00 (0·93, 4·30) | |

2. Controls matched for year of birth

| | Length of service (years) | | | | | | | | | |
|---|---|---|---|---|---|---|---|---|---|---|
| | 0–4 | | 5–9 | | 10–14 | | 15+ | | Total | |
| Exposure | Case | Control | Case | Control | Case | Control | Case | Control | Case | Control |
| Low | 7 | 20 | 3 | 17 | 5 | 8 | 3 | 27 | 18 | 72 |
| Medium or high | 1 | 2 | 4 | 3 | 6 | 8 | 7 | 23 | 18 | 36 |
| RR (95% confidence interval) | 1·43 (0·11, 18·32) | | 7·56 (1·09, 52·38) | | 1·20 (0·26, 5·59) | | 2·74 (0·63, 11·83) | | 2·00 (0·93, 4·30) | |

the time of the occurrence (death) of their related case, and only 9% had died more than 5 years before their related case.

To match living controls with dead cases might introduce bias as, by definition, the controls could not have had a fatal illness and died on the "same" date. It would have been desirable to match with controls alive at the date of first development of leukaemia for cases, but this information was not known for our subjects.

Misclassification of information on exposure variables or disease states could produce spuriously high or low measures of effect. The completeness and detail of the job histories on which the exposure to benzene was based depended on (a) the personnel records of the companies in the 8 different refineries, (b) the type of main occupation, and to a lesser extent (c) the period of time concerned. Lack of documented data on environmental benzene exposure over the years covered by the study meant that knowledge of the exposure history of the refineries and plants had to depend on the memories of the occupational hygienists in the working party. This necessitated a fairly crude ordinal classification of exposure to benzene, with no attempt at quantification of degree of exposure for different jobs.

In deciding whether to allocate men to the medium- or high-exposure categories, the length of service on the job was sometimes taken into account in addition to the type of occupation. There was no "double counting" of length of service in those controls who were matched for year of birth and length of service, as these two exposure categories were in fact combined in the final analyses. Length of service was not considered in deciding whether to allocate men to the low-exposure category.

A misleading measure of risk may be obtained if the factor under investigation is confounded with another variable of interest. Confounding variables may be taken into account (i) in the design stage by matching, and (ii) in the analysis either by stratification or by inclusion in a linear logistic model.

Two sets of controls were used in this study (a) matched for year of birth and length of service and (b) matched for year of birth. The analyses of both control sets, using the method described by Pike & Morrow (1970), showed a weak association between benzene exposure and leukaemia. The fitting of the logistic model showed an association of benzene exposure with leukaemia for control set (a). When the logistic model was fitted for control set (b) taking into account length of service, the relative risk of benzene exposure was significantly different from unity at the 5% level.

The results obtained from the analyses of this case-control study thus indicate an increase in the risk of leukaemia in the group whose exposure to benzene was assessed as medium or high. There was no evidence that the risk increased with length of service.

However, the exact nature of the relationship between benzene exposure and leukaemia is not readily expressed when one considers the results from (a) the examination of the mortality patterns of 35,000 workers in 8 refineries, and (b) this case-control study. There was no overall excess of deaths from leukaemia in the workforce as a whole, when compared with that expected based on national rates (O = 30, E = 31·96, $P = 0{\cdot}41$). Examination of the types of leukaemia was handicapped, as the source of information was death certificates and the numbers were small. However, there was no excess of deaths from the cytological types of leukaemia which have been shown in previous studies to be particularly associated with benzene exposure (Aksoy *et al.*, 1976; Environmental Protection Agency, 1977). It was not possible to calculate expected values using national mortality rates for the different exposure groups, as the exposure levels were not known for the total refinery study population. Taking the above points into consideration, if there were an increased risk of leukaemia due to benzene exposure, it could have only been one that affected a very small proportion of men within the refinery workforce.

The data for this study was handled by Mrs Carol Fair and Ms Deborah Cummings in the research team and the typing was carried out by Mrs Vera Watson. The basic information was abstracted by staff in personnel departments at the refineries. We are grateful to the Office of Population, Censuses and Surveys and the Registrar General for Scotland for provision of the death details. A working party from the oil industry helped with the assessment of benzene exposure levels. We are grateful to Peter G. Smith for providing us with the computer program to fit the logistic model and for his advice on the interpretation of the results. The Division of Epidemiology acknowledges financial support from the Cancer Research Campaign.

### REFERENCES

AKSOY, M., DINCOL, K., ERDEM, S. & DINCOL, G. (1972) Acute leukaemia due to chronic exposure to benzene. *Am. J. Med.*, **52,** 160.

AKSOY, M., ERDEM, S. & DINCOL, G. (1976) Types of leukaemia in chronic benzene poisoning. A study in thirty-four patients: *Acta Haematol.*, **55,** 65.

AKSOY, M., ERDEM, S. & DINCOL, G. (1974) Leukaemia in shoe-workers exposed chronically to benzene. *Blood*, **44,** 837.

ARMITAGE, P. (1974) *Statistical Methods in Medical Research.* Oxford: Blackwell.

BRESLOW, N. E., DAY, N. E., HALVORSON, K. T., PRENTICE, R. L. & SABAI, C. (1978) Estimation of multiple relative risk functions in matched case-control studies. *Am. J. Epidemiol.*, **108,** 299.

COX, D. R. (1970) *Analysis of Binary Data.* London & Hall.

ENVIRONMENTAL PROTECTION AGENCY (1977) *Benzene Health Effects Assessment. Section* 4, *Benzene Toxicity in Man.* Washington: E.P.A.

GOLDSTEIN, B. D. (1977) Haematotoxicity in humans, Chapter 7. In *Benzene Toxicity: a Critical Evaluation. J. Toxicol. Environ. Health*, Suppl. 2.

HANIS, N. M., STAVIAKY, K. M. & FOWLER, J. L. (1979) Cancer mortality in oil refinery workers. *J. Occup. Med.*, **21,** 167.

HOLFORD, T. R., WHITE, C. & KELSEY, J. L. (1978) Multivariate analysis for matched case-control studies. *Am. J. Epidemiol.*, **107,** 245.

INFANTE, P. F., RINSKY, R. A., WAGONER, J. K. & YOUNG, R. J. (1977) Leukaemia in benzene workers. *Lancet*, ii, 76.

MANCUSO, T. E., CIOCCO, A. & EL-ALTAR, A. A. (1968) An epidemiological approach to the rubber industry. *J. Occup. Med.*, **10,** 213.

MANTEL, N. (1973) Synthetic retrospective studies and related topics. *Biometrics*, **29,** 479.

MCDERMOTT, H. J. & VOS, G. A. (1979) Service station attendants' exposure to benzene and gasoline vapours. *Am. Ind. Hyg. Ass. J.*, **40,** 315.

MCMICHAEL, A. J., SPIRTAS, R., KUPPER, I. L. & GAMBLE, J. F. (1975) Solvent exposure and leukaemia among rubber workers: An epidemiologic study. *J. Occup. Med.*, **17,** 234.

MIETTINEN, O. (1974) Confounding and effect modification. *Am. J. Epidemiol.*, **100,** 350.

PANDYA, K. P., RAO, G. S., DHASMANA, A. & ZAIDI, S. H. (1975) Occupational exposure of petrol pump workers. *Ann. Occup. Hyg.*, **18,** 363.

PARKINSON, G. S. (1971) Benzene in motor gasoline —an investigation into possible health hazards in and around filling stations and in normal transport operations. *Ann. Occup. Hyg.*, **14,** 145.

PIKE, M. C. & MORROW, R. H. (1970) Statistical analysis of patient-control studies in epidemiology. Factor under investigation an all-or-none variable. *Br. J. Prev. Soc. Med.*, **24,** 42.

PRENTICE, R. (1976) Use of the logistic model in retrospective studies. Biometrics, **32,** 599.

REDMOND, C. K., CIOCCO, A., LLOYD, J. W. & RUSH, H. W. (1972) Long term mortality study of steel workers. VI Mortality from malignant neoplasms among coke oven workers. *J. Occup. Med.*, **14,** 621.

Rushton, L. & Alderson, M. R. (1980) An epidemiological survey of eight oil refineries in the U.K.—Final report. *Institute of Petroleum.*

Sherwood, R. J. (1971) Occupational hygiene in aromatic plants. *Ann. Occup. Hyg.*, **14,** 125.

Sherwood, R. J. (1972) Evaluation of exposure to benzene vapour during the loading of petrol. *Br. J. Ind. Med.*, **29,** 65.

Smith, P. G., Pike, M. C., Hill, A. P., Breslow, N. E. & Day, N. E. (1979) Multivariate conditional logistic analysis of stratum-matched case-control studies.

Tabershaw, I. R. (1974) A mortality study of petroleum refinery workers. *API Project OH-1 No. 129.*

Theriault, G. & Goulet, L. (1979) A mortality study of oil refinery workers. *J. Occup. Med.*, **21,** 367.

Thomas, T. L., Decoufle, P. & Moure-Eraso, M. S. (1980) Mortality among workers employed in petroleum refining and petrochemical plants. *J. Occup. Med.*, **22,** 97.

Thorpe, J. J. (1974) Epidemiologic survey of leukaemia in persons potentially exposed to benzene. *J. Occup. Med.*, **16,** 375.

Vigliani, E. C. & Saita, G. (1964) Benzene and leukaemia. *N. Eng. J. Med.*, **271,** 872.

Vigliani, E. C. & Forni, A. (1976) Benzene and leukaemia. *Env. Res.*, **11,** 122.

Vitaliano, P. P. (1978) The use of logistic repression for modelling risk factors: With application to non-melanoma skin cancer. *Am. J. Epidemiol.*, **108,** 402.

Wright, N. H., Vessey, M. P. & Kenward, B. (1978) Neoplasia and dysplasia of the cervix uteri and contraception: A possible protective effect of the diaphragm. *Br. J. Cancer*, **38,** 273.